# An indirect ELISA for the detection of antibodies against *Dirofilaria* spp. in cats

**DOI:** 10.1186/s13071-024-06657-z

**Published:** 2025-01-19

**Authors:** Lívia Perles, Mariaelisa Carbonara, Jairo Alfonso Mendoza-Roldan, Luigi Venco, Simona Gabrielli, Domenico Otranto

**Affiliations:** 1 Department of Veterinary Medicine, University of Bari, Bari, Italy; 2 Ospedale Veterinario Città Di Pavia, Pavia, Italy; 3 Department of Public Health and Infectious Diseases “Sapienza”, University of Rome, Rome, Italy; 4 Department of Veterinary Clinical Sciences, City University of Hong Kong, Hong Kong, China

**Keywords:** *Dirofilaria immitis*, *Dirofilaria repens*, Serology, Somatic antigens, Feline dirofilariosis

## Abstract

**Background:**

*Dirofilaria immitis* and *D. repens* are mosquito-borne filaroids that primarily infect dogs but also cats. Diagnosing feline dirofilariosis is challenging because of the low parasitic burdens and transient or absent microfilaremia. To improve detection of antibodies against *Dirofilaria* spp. in cats, an indirect enzyme-linked immunosorbent assay (ELISA) using somatic antigens of *D. immitis* was standardized.

**Methods:**

Serum samples from cats positive for *D. immitis*, *D. repens*, bronchopulmonary metastrongylids and gastrointestinal helminths as well as negative sera were tested to evaluate the sensitivity (Se) and specificity (Sp). Three different antigen concentrations (2, 3 and 4 μg/ml) and two concentrations of horseradish peroxidase (HRP) IgG anti-cat conjugate (1:10,000 and 1:20,000) were used to screen the optimal titration of the test. Once the best conditions were established, Se and Sp were assessed by testing 151 serum samples positive for *D. immitis*, *D. repens* and other parasites (i.e. feline lungworms, gastrointestinal helminths) or negative.

**Results:**

The optimized ELISA showed 89% Se and 98% Sp in detecting *D. immitis* infections, presenting a cross-reactivity with *D. repens*. A cut-off point of 1.150 was established to classify positive and negative samples.

**Conclusions:**

The developed ELISA may improve the detection of antibodies against *Dirofilaria* spp. in cats, also in epidemiological contexts characterized by multiple helminth infections. Future efforts will focus on reducing the cross-reactivity with *D. repens*, therefore increasing ELISA Sp.

**Graphical Abstract:**

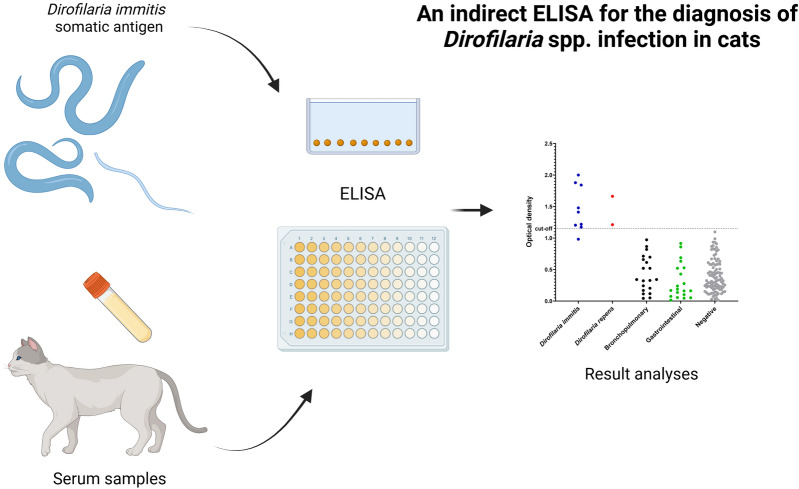

**Supplementary Information:**

The online version contains supplementary material available at 10.1186/s13071-024-06657-z.

## Background

*Dirofilaria immitis* and *D. repens* are mosquito-borne filarial parasites, which infect primarily dogs but also cats, as well as other wild carnivores and humans [1, 2]. While the first species is globally known as the causative agent of heartworm disease in dogs [1], the second has been less investigated because of limited clinical relevance due to the subcutaneous localization [1, 2]. The diagnosis of *Dirofilaria* spp. infection relies on either direct detection of microfilariae (mf) in blood by Knott’s test, followed by morphological/molecular identification, or *D. immitis* antigens by commercial enzyme-linked immunosorbent assay (ELISA). Specifically, the diagnosis of acute feline cardiopulmonary dirofilariosis should not be based solely on the results of diagnostic laboratory tests, as these may yield false-negative results, especially during the early stages of the infection. Complete anamnesis, physical examination and blood cell count are crucial for a correct interpretation of diagnostic laboratory test results [3, 4]. In addition, adult forms of *D. immitis* may be observed by imaging investigations (e.g. echography or radiographic) in the heart or pulmonary arteries as well as *D. repens* nematodes in subcutaneous nodules [3].

Regarding feline dirofilariosis, *D. immitis*-infected animals may present severe respiratory manifestations with potential *exitus* [5–7], while those infected by *D. repens* are mostly asymptomatic [8–10]. Cats, unlike dogs, exhibit a low parasitic burden, with none or transient microfilaremia (i.e. occult infections) [11, 12]. The underlying mechanisms for the differences in infection patterns between cats and dogs remain unclear, although it might be because of an inherent resistance to *D. immitis* and/or a protective immune response in cats [12]. Given the above, direct parasite detection in cats represents a complex task. In the absence of circulating mf and female antigens, antibody detection indicates the exposure of cats to *Dirofilaria* spp. and thus to the infection in animals living in or traveling to endemic regions [5]. The specificity (Sp) of antibody-based tests could be impaired by the simultaneous occurrence of other endoparasites (i.e. bronchopulmonary and gastrointestinal helminths), which usually infect cats [13], as demonstrated for the diagnosis of *Aelurostrongylus abstrusus* by an ELISA test [14]. Under the above circumstances, *D. immitis* antibody-based ELISA showed a higher positivity rate compared to antigen-based tests (i.e. 9.4% vs 0.5%, [15]), challenging the interpretation of diagnostic results, mainly in areas where both *D. immitis* and *D. repens* infect cats. Therefore, we aimed to develop an ELISA based on *D. immitis* somatic antigens to evaluate its sensitivity (Se) and Sp to detect antibodies, considering potential cross-reactions with other nematodes.

## Methods

### Somatic antigen preparation

*Dirofilaria immitis* somatic antigens (DiSA) were prepared from a total of 20 adult nematode specimens (males and females) collected in the frame of previous studies (see [16]). Nematodes were washed three times in a 1% PBS solution and then suspended in a lysis buffer (8 M urea, 4% CHAPS, 40 mM Trizma base), supplemented with protease inhibitor cocktail (Roche, Berlin, Germany). The protein extract was then homogenized in a glass Potter homogenizer and disintegrated by sonication at 70 kHz three times for 30 s in sterile saline solution. The homogenate was centrifuged at 16,000 × g for 30 min. The supernatant was dialyzed against 0.01 M PBS, pH 7.2. The antigen concentration was measured using the Bradford method [17], adjusted to 4.5 µg/µl in the final solution and cryopreserved at – 20 °C until required.

### Assay standardization

To screen the optimal initial ELISA conditions, a serum sample from a cat positive for *D. immitis* by qPCR [18] and SNAP^™^ test (Canine Heartworm PF, IDEXX Laboratories Inc., MA, USA) was tested along with positive samples for other helminth infections (*n* = 5; i.e. *n* = 2 bronchopulmonary, *n* = 2 gastrointestinal helminths and *n* = 1 *D. repens*) and negative for any of the above (*n* = 2). All samples were tested in duplicate. Optical density of the positive and negative samples was assessed based on testing three different concentrations of somatic antigen (2, 3 and 4 μg/ml) and two concentrations of horseradish peroxidase (HRP) IgG anti-cat conjugate (1:10,000 and 1:20,000). Initially, plates were coated at 200 µl per well with the three selected DiSA concentrations, diluted in carbonate buffer (pH 9.6) and incubated overnight at 4 °C. The plates were subsequently rinsed three times with 200 μl PBS-Tween20 0.5% under continuous shaking at 300 rpm at 37 °C for 30 min, thereby eliminating unbound antigens. Plates were blocked with 200 μl Blocking Reagent (Roche Diagnostics, Mannheim, Germany, GmBH) at 37 °C, 300 rpm for 1 h and 30 min and washed three times with PBS-Tween20 0.5% and dried. Then, 100 μl of all selected samples (i.e. *D. immitis*, bronchopulmonary, gastrointestinal and *D. repens* positive sera; negative ones) were subjected to dilution in homemade ELISA diluent (1.7 g NaCl, 0.426 g Na₂HPO₄, 0.078 g NaH₂PO₄, 2 g bovine serum albumin, 500 μl Tween20) at 1:200 concentration and incubated for 1 h at 37 °C and 300 rpm. Plates were washed three times with 200 μl PBS-Tween20 0 and 5% and, once completely dried, were incubated with 100 μl of HRP IgG anti-cat conjugate (goat anti-feline IgG, Sigma-Aldrich, St. Louis, MO, USA) in the two above-mentioned dilutions at 37 °C and 300 rpm for 1 h. After washing and drying, plates were incubated with 100 μl of TMB chromogen solution (tetramethyl benzidine, Sigma-Aldrich) for 7 min at room temperature. The colorimetric reaction was terminated with 50 μl stop solution (Invitrogen, Thermo Fisher Scientific, Vienna, Austria). The plate was then read using Absorbance 96 Plate reader Enzo (Byonoy, Hamburger, Germany) at a wavelength (λ) of 450 nm and a reference filter at a λ of 620 nm.

### ELISA validation with field cat sera

Once established, the best conditions for the assay (i.e. antigen and conjugate concentrations), Se and Sp were assessed by testing 151 cat serum samples, including sera of *D. immitis*-positive cats (*n* = 9), cats (*n* = 20) positive for feline lungworm antibodies [14], cats (*n* = 20) positive for gastrointestinal helminths (i.e. *Toxocara cati*, *Taenia* sp., *Mesocestoides* sp.) (unpublished data) and cats (*n* = 2) positive for *D. repens* at qPCR (unpublished data). Sera of negative cats (*n* = 100) coming from non-endemic areas that tested molecularly or antigenically negative for *Dirofilaria* spp. infections were also included as negative controls. The diagnostic Se and Sp were calculated by plotting the receiver-operating characteristic (ROC) curve [19]. The cut-off point was established by calculating the mean value ± 3 standard deviations (3SD) of the 100 negative controls.

## Results

### Assay standardization

Results of ELISA standardization are shown as mean OD in Table 1. The OD values of *D. immitis*-positive control sample were similar across the three antigen dilutions (2, 3 and 4 µg/ml), with a variation of approximately 1.5 OD unit by the two conjugate dilutions (1:10,000 and 1:20,000). Negative control samples displayed low average OD values at all the dilutions, with the lowest OD records (0.206) with 2 µg/ml of antigen and 1:20,000 of conjugate and the highest (0.594) with 4 µg/ml of antigen and 1:10,000 of conjugate. For *D. repens*-positive samples, the highest conjugate dilution (1:20,000) revealed a decrease of approximately 1.96 OD units compared to the lowest conjugate dilution (1:10,000). Minor variations in OD values were recorded between antigen concentrations (approximately 0.2–0.4 OD).

**Table 1:** Optical density mean values obtained during ELISA assay standardization by testing serum samples positive for *Dirofilaria immitis*, *D. repens*, bronchopulmonary metastrongylids, gastrointestinal helminths and negative samples, with three different antigen concentrations (2, 3 and 4 μg/ml) and two different IgG anti-cat conjugate concentrations (1:10,000 and 1:20,000)

	No. samples	2 µg/ml		3 µg/ml		4 µg/ml	
		1:10,000	1:20,000	1:10,000	1: 20,000	1:10,000	1:20,000
*Dirofilaria immitis*	1	3.903	2.209	3.830	2.168	3.907	2.225
*Dirofilaria repens*	1	3.342	1.612	2.689	1.769	3.572	1.676
Bronchopulmonary	2	1.458	0.578	1.135	0.817	1.068	0.649
Gastrointestinal	2	0.953	0.447	1.036	0.467	1.072	0.649
Negative	2	0.553	0.206	0.556	0.330	0.594	0.252

Similarly, for bronchopulmonary and gastrointestinal positive samples, OD variations according to antigen concentrations were minor, while at the 1:20,000 conjugate dilution, OD values were consistently low (Table 1).

Based on the above, the best condition selected and used in the following for the ELISA assay was 2 µg/ml *D. immitis* antigen and 1:20,000 IgG anti-cat conjugate (Table 1).

### ELISA validation using field cat sera

**Fig. 1:**
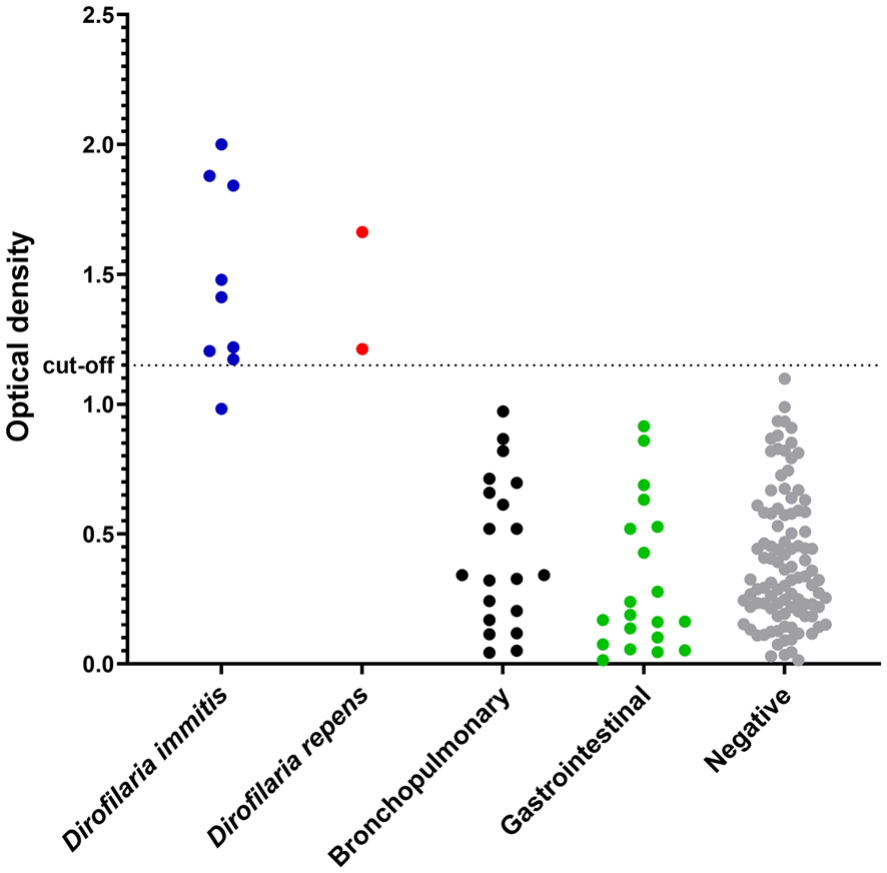
Optical density (OD) obtained for all examined sera (i.e. positive for *Dirofilaria immitis, D. repens*, bronchopulmonary and gastrointestinal helminths as well as negative) according to the established cut-off (1.15 OD)

The mean OD value for positive *D. immitis* samples (*n* = 9) was 1.465 ± 0.341 (range, 0.982 to 2) and for negative samples (*n* = 100) 0.401 ± 0.273 (range, 0.014 to 1.098). OD results for the two *D. repens*-positive samples were 1.479 and 1.663. Bronchopulmonary and gastrointestinal samples were all negative (Fig. 1; Table S1). The correspondence between ELISA positivity and results retrieved by other *Dirofilaria* spp. tests (i.e. qPCR and SNAP test) is reported in Supplementary Table 2. The ROC curve analysis revealed that at the threshold of 0.5 OD, Se was 100%, indicating that all positive samples were correctly identified, with a low Sp (66%). As the threshold increased to 0.8, Se remained at 100%, while Sp improved to 87%. At thresholds of 0.9 and 1.0, sensitivity decreased to 90%, yet Sp rose to 94%. The positive predictive value (PPV) increased from 16% at 0.5 to 80% at 1.2, indicating enhanced reliability in positive results as the threshold was adjusted, whereas the negative predictive value (NPV) remained consistently high across all thresholds (Table 2). The cut-off established was 1.150, with 89% Se and 98% Sp.

**Table 2:** Receiver-operating characteristic (ROC) data for ELISA validation using field cat sera

Threshold	Sensitivy (%)	Specificity (%)	PPV (%)	NPV (%)
0.5	100	66	16	100
0.6	100	77	21	100
0.7	100	85	29	100
0.8	100	87	33	100
0.9	90	94	50	99
1.0	90	94	50	99
1.1	89	98	73	99
1.2	89	99	80	99

*PPV*: positive predictive value, *NPV*: negative predictive value

## Discussion

The indirect ELISA standardized herein represents a novel serological procedure for detecting antibodies against *Dirofilaria* spp. in cats, with *D. immitis* somatic antigens demonstrating a high Se in detecting exposed animals and Sp within the genus *Dirofilaria* spp. Overall, data highlight the importance of refining assay parameters (e.g. antigen concentration and conjugate dilution) to obtain proper test performances in terms of Se and Sp, rendering the ELISA suitable for clinical and epidemiological purposes.

Furthermore, our study reinforces the suitability of somatic antigens for the indirect diagnosis of *Dirofilaria* spp. exposition in cats living in areas where polyparasitism by feline lungworms and gastrointestinal helminths may occur [13]. Accordingly, the above test overcomes the cross-reactions between *Dirofilaria* spp. and other helminths [14, 20].

ELISA has been previously applied to diagnose feline dirofilariosis by using either *D. immitis* synthetic peptides or somatic/excretory antigens (i.e. in-house methodology). In the first case, a strong and specific IgG response against *D. immitis* antigens was confirmed in experimentally infected cats by ELISA based on a pool of six synthetic peptides (i.e. coating with 0.8 μg of the peptide pool, 1:4000 conjugate dilution and 0.5 OD cut-off) [21]. The same ELISA was used for epidemiological studies, providing an average seroprevalence from 9.4% to 12% in Spain and Italy, respectively [15, 22]. In both studies above, possible cross-reactions due to polyparasitism were not assessed. Again, the ELISA based on somatic antigens was effective in detecting IgG response in naturally infected cats, with 10 of the 12 cats examined positive to heartworms in echography [16]. In the latter study, at the ELISA condition employed (i.e. coating with 4 μg somatic antigen, 1:800 cat serum dilution and 1:4000 conjugate dilution), cross-reactions with *T. cati* were excluded, whereas bronchopulmonary metastrongylids were not serologically ruled out. Here, we used a lower concentration of somatic antigen (0.4 µg) along with a higher conjugate dilution (1:20,000), providing an ELISA assay capable of detecting eight out of nine infected cats with a Sp of 98%. A limitation of the present study is the small number of *Dirofilaria* spp.-positive cats due to the difficulty in obtaining dirofilariasis-infected cats. In addition, considering that different *Dirofilaria* spp. strains are known to circulate in Europe [23, 24], antigens for the above ELISA should be prepared at country/regional level.

The observed cross-reactivity between *D. immitis* and *D. repens* confirms that somatic antigens are not species-specific, as many proteins are shared within the species of the *Dirofilaria* genus [25–27]. Nonetheless, the low number of *D. repens*-positive samples tested prevents drawing definitive conclusions about their usefulness in diagnosing this infection. Both the duration and time of onset of antibody production need to be assessed in longitudinal investigations aiming to follow up *Dirofilaria* spp.-positive cats.

## Conclusions

The optimized indirect ELISA developed here improves the detection of antibodies against *Dirofilaria* spp. in cats. Results demonstrated that high secondary antibody dilutions (1:20,000) are crucial for minimizing false-positive results, coming from bronchopulmonary and gastrointestinal infections, maintaining appropriate OD values for true-positive samples. Future studies should focus on reducing the cross-reactivity between both *Dirofilaria* spp., promoting the test usefulness for both clinical and research purposes.

## Supplementary Information

Supplementary material 1. Table S1. Optical density values of cat serum samples tested, including negative animals and positive to *Dirofilaria immitis*, *Dirofilaria repens*, *Aelurostrongylus abstrusus* and gastrointestinal helminthswith thresholds from 0.5 to 1.2 OD used to calculate the receiver operating characteristiccurve. Table S2. ELISA positivity according to results retrieved by other *Dirofilaria* spp. tests

## Data Availability

All data supporting the findings of this study are available within the paper and its Supplementary Information.
