# Supplementary material for: An indirect ELISA for the detection of antibodies against Dirofilaria spp. in cats
Source: Parasit Vectors. 2025 Jan 19;18:16. doi: 10.1186/s13071-024-06657-z (PMC11744980; doi:10.1186/s13071-024-06657-z)
Supplement: Supplementary file 1 — Supplementary material 1. Table S1. Optical density values of cat serum samples tested, including negative animals and positive to Dirofilaria immitis, Dirofilaria repens, Aelurostrongylus abstrusus and gastrointestinal helminthswith thresholds from 0.5 to 1.2 OD used to calculate the receiver operating characteristiccurve. Table S2. ELISA positivity according to results retrieved by other Dirofilaria spp. tests [file 13071_2024_6657_MOESM1_ESM.docx]

**Table S1.** Optical density values of cat serum samples tested, including negative animals and positive to *Dirofilaria immitis*, *Dirofilaria repens*, *Aelurostrongylus abstrusus* and gastrointestinal helminths (i.e., *Toxocara cati*, *Taenia* sp., *Mesocestoides lineatus* and *Dipylidium caninum*) with thresholds from 0.5 to 1.2 OD used to calculate the receiver operating characteristic (ROC) curve.

| **Sample** | **OD** | **Parasitological**  **status** | **Threshold 0.5** | **Threshold 0.6** | **Threshold 0.7** | **Threshold 0.8** | **Threshold 0.9** | **Threshold 1.0** | **Threshold 1.1** | **Threshold 1.2** |
| --- | --- | --- | --- | --- | --- | --- | --- | --- | --- | --- |
| **1** | 0,273 | Negative | Negative | Negative | Negative | Negative | Negative | Negative | Negative | Negative |
| **2** | 0,23 | Negative | Negative | Negative | Negative | Negative | Negative | Negative | Negative | Negative |
| **3** | 0,989 | Negative | Positive | Positive | Positive | Positive | Positive | Negative | Negative | Negative |
| **4** | 0,268 | Negative | Negative | Negative | Negative | Negative | Negative | Negative | Negative | Negative |
| **5** | 0,09 | Negative | Negative | Negative | Negative | Negative | Negative | Negative | Negative | Negative |
| **6** | 0,332 | Negative | Negative | Negative | Negative | Negative | Negative | Negative | Negative | Negative |
| **7** | 0,868 | Negative | Positive | Positive | Positive | Positive | Negative | Negative | Negative | Negative |
| **8** | 0,23 | Negative | Negative | Negative | Negative | Negative | Negative | Negative | Negative | Negative |
| **9** | 0,935 | Negative | Positive | Positive | Positive | Positive | Positive | Negative | Negative | Negative |
| **10** | 0,792 | Negative | Positive | Positive | Positive | Negative | Negative | Negative | Negative | Negative |
| **11** | 0,144 | Negative | Negative | Negative | Negative | Negative | Negative | Negative | Negative | Negative |
| **12** | 1,098 | Negative | Positive | Positive | Positive | Positive | Positive | Positive | Positive | Negative |
| **13** | 0,598 | Negative | Positive | Negative | Negative | Negative | Negative | Negative | Negative | Negative |
| **14** | 0,399 | Negative | Negative | Negative | Negative | Negative | Negative | Negative | Negative | Negative |
| **15** | 0,184 | Negative | Negative | Negative | Negative | Negative | Negative | Negative | Negative | Negative |
| **16** | 0,668 | Negative | Positive | Positive | Negative | Negative | Negative | Negative | Negative | Negative |
| **17** | 0,59 | Negative | Positive | Negative | Negative | Negative | Negative | Negative | Negative | Negative |
| **18** | 0,249 | Negative | Negative | Negative | Negative | Negative | Negative | Negative | Negative | Negative |
| **19** | 0,151 | Negative | Negative | Negative | Negative | Negative | Negative | Negative | Negative | Negative |
| **20** | 0,828 | Negative | Positive | Positive | Positive | Positive | Negative | Negative | Negative | Negative |
| **21** | 0,44 | Negative | Negative | Negative | Negative | Negative | Negative | Negative | Negative | Negative |
| **22** | 0,214 | Negative | Negative | Negative | Negative | Negative | Negative | Negative | Negative | Negative |
| **23** | 0,452 | Negative | Negative | Negative | Negative | Negative | Negative | Negative | Negative | Negative |
| **24** | 0,219 | Negative | Negative | Negative | Negative | Negative | Negative | Negative | Negative | Negative |
| **25** | 0,573 | Negative | Positive | Negative | Negative | Negative | Negative | Negative | Negative | Negative |
| **26** | 0,244 | Negative | Negative | Negative | Negative | Negative | Negative | Negative | Negative | Negative |
| **27** | 0,578 | Negative | Positive | Negative | Negative | Negative | Negative | Negative | Negative | Negative |
| **28** | 0,631 | Negative | Positive | Positive | Negative | Negative | Negative | Negative | Negative | Negative |
| **29** | 0,337 | Negative | Negative | Negative | Negative | Negative | Negative | Negative | Negative | Negative |
| **30** | 0,359 | Negative | Negative | Negative | Negative | Negative | Negative | Negative | Negative | Negative |
| **31** | 0,074 | Negative | Negative | Negative | Negative | Negative | Negative | Negative | Negative | Negative |
| **32** | 0,124 | Negative | Negative | Negative | Negative | Negative | Negative | Negative | Negative | Negative |
| **33** | 0,669 | Negative | Positive | Positive | Negative | Negative | Negative | Negative | Negative | Negative |
| **34** | 0,933 | Negative | Positive | Positive | Positive | Positive | Positive | Negative | Negative | Negative |
| **35** | 0,531 | Negative | Positive | Negative | Negative | Negative | Negative | Negative | Negative | Negative |
| **36** | 0,821 | Negative | Positive | Negative | Negative | Negative | Negative | Negative | Negative | Negative |
| **37** | 0,288 | Negative | Negative | Negative | Negative | Negative | Negative | Negative | Negative | Negative |
| **38** | 0,509 | Negative | Positive | Negative | Negative | Negative | Negative | Negative | Negative | Negative |
| **39** | 0,233 | Negative | Negative | Negative | Negative | Negative | Negative | Negative | Negative | Negative |
| **40** | 0,323 | Negative | Negative | Negative | Negative | Negative | Negative | Negative | Negative | Negative |
| **41** | 0,852 | Negative | Positive | Positive | Positive | Positive | Negative | Negative | Negative | Negative |
| **42** | 0,464 | Negative | Negative | Negative | Negative | Negative | Negative | Negative | Negative | Negative |
| **43** | 0,392 | Negative | Negative | Negative | Negative | Negative | Negative | Negative | Negative | Negative |
| **44** | 0,638 | Negative | Positive | Positive | Negative | Negative | Negative | Negative | Negative | Negative |
| **45** | 0,254 | Negative | Negative | Negative | Negative | Negative | Negative | Negative | Negative | Negative |
| **46** | 0,323 | Negative | Negative | Negative | Negative | Negative | Negative | Negative | Negative | Negative |
| **47** | 0,127 | Negative | Negative | Negative | Negative | Negative | Negative | Negative | Negative | Negative |
| **48** | 0,109 | Negative | Negative | Negative | Negative | Negative | Negative | Negative | Negative | Negative |
| **49** | 0,293 | Negative | Negative | Negative | Negative | Negative | Negative | Negative | Negative | Negative |
| **50** | 0,812 | Negative | Positive | Positive | Positive | Positive | Negative | Negative | Negative | Negative |
| **51** | 0,153 | Negative | Negative | Negative | Negative | Negative | Negative | Negative | Negative | Negative |
| **52** | 0,61 | Negative | Positive | Positive | Negative | Negative | Negative | Negative | Negative | Negative |
| **53** | 0,363 | Negative | Negative | Negative | Negative | Negative | Negative | Negative | Negative | Negative |
| **54** | 0,22 | Negative | Negative | Negative | Negative | Negative | Negative | Negative | Negative | Negative |
| **55** | 0,675 | Negative | Positive | Positive | Negative | Negative | Negative | Negative | Negative | Negative |
| **56** | 0,579 | Negative | Positive | Negative | Negative | Negative | Negative | Negative | Negative | Negative |
| **57** | 0,726 | Negative | Positive | Positive | Positive | Negative | Negative | Negative | Negative | Negative |
| **58** | 0,583 | Negative | Positive | Negative | Negative | Negative | Negative | Negative | Negative | Negative |
| **59** | 0,405 | Negative | Negative | Negative | Negative | Negative | Negative | Negative | Negative | Negative |
| **60** | 0,454 | Negative | Negative | Negative | Negative | Negative | Negative | Negative | Negative | Negative |
| **61** | 0,112 | Negative | Negative | Negative | Negative | Negative | Negative | Negative | Negative | Negative |
| **62** | 0,819 | Negative | Positive | Positive | Positive | Positive | Negative | Negative | Negative | Negative |
| **63** | 0,094 | Negative | Negative | Negative | Negative | Negative | Negative | Negative | Negative | Negative |
| **64** | 0,469 | Negative | Negative | Negative | Negative | Negative | Negative | Negative | Negative | Negative |
| **65** | 0,585 | Negative | Positive | Negative | Negative | Negative | Negative | Negative | Negative | Negative |
| **66** | 0,247 | Negative | Negative | Negative | Negative | Negative | Negative | Negative | Negative | Negative |
| **67** | 0,116 | Negative | Negative | Negative | Negative | Negative | Negative | Negative | Negative | Negative |
| **68** | 0,27 | Negative | Negative | Negative | Negative | Negative | Negative | Negative | Negative | Negative |
| **69** | 0,374 | Negative | Negative | Negative | Negative | Negative | Negative | Negative | Negative | Negative |
| **70** | 0,262 | Negative | Negative | Negative | Negative | Negative | Negative | Negative | Negative | Negative |
| **71** | 0,193 | Negative | Negative | Negative | Negative | Negative | Negative | Negative | Negative | Negative |
| **72** | 0,443 | Negative | Negative | Negative | Negative | Negative | Negative | Negative | Negative | Negative |
| **73** | 0,142 | Negative | Negative | Negative | Negative | Negative | Negative | Negative | Negative | Negative |
| **74** | 0,879 | Negative | Positive | Positive | Positive | Positive | Negative | Negative | Negative | Negative |
| **75** | 0,313 | Negative | Negative | Negative | Negative | Negative | Negative | Negative | Negative | Negative |
| **76** | 0,445 | Negative | Negative | Negative | Negative | Negative | Negative | Negative | Negative | Negative |
| **77** | 0,14 | Negative | Negative | Negative | Negative | Negative | Negative | Negative | Negative | Negative |
| **78** | 0,443 | Negative | Negative | Negative | Negative | Negative | Negative | Negative | Negative | Negative |
| **79** | 0,408 | Negative | Negative | Negative | Negative | Negative | Negative | Negative | Negative | Negative |
| **80** | 0,325 | Negative | Negative | Negative | Negative | Negative | Negative | Negative | Negative | Negative |
| **81** | 0,234 | Negative | Negative | Negative | Negative | Negative | Negative | Negative | Negative | Negative |
| **82** | 0,443 | Negative | Negative | Negative | Negative | Negative | Negative | Negative | Negative | Negative |
| **83** | 0,909 | Negative | Positive | Positive | Positive | Positive | Positive | Negative | Negative | Negative |
| **84** | 0,184 | Negative | Negative | Negative | Negative | Negative | Negative | Negative | Negative | Negative |
| **85** | 0,029 | Negative | Negative | Negative | Negative | Negative | Negative | Negative | Negative | Negative |
| **86** | 0,301 | Negative | Negative | Negative | Negative | Negative | Negative | Negative | Negative | Negative |
| **87** | 0,421 | Negative | Negative | Negative | Negative | Negative | Negative | Negative | Negative | Negative |
| **88** | 0,132 | Negative | Negative | Negative | Negative | Negative | Negative | Negative | Negative | Negative |
| **89** | 0,304 | Negative | Negative | Negative | Negative | Negative | Negative | Negative | Negative | Negative |
| **90** | 0,503 | Negative | Positive | Negative | Negative | Negative | Negative | Negative | Negative | Negative |
| **91** | 0,218 | Negative | Negative | Negative | Negative | Negative | Negative | Negative | Negative | Negative |
| **92** | 0,014 | Negative | Negative | Negative | Negative | Negative | Negative | Negative | Negative | Negative |
| **93** | 0,045 | Negative | Negative | Negative | Negative | Negative | Negative | Negative | Negative | Negative |
| **94** | 0,183 | Negative | Negative | Negative | Negative | Negative | Negative | Negative | Negative | Negative |
| **95** | 0,202 | Negative | Negative | Negative | Negative | Negative | Negative | Negative | Negative | Negative |
| **96** | 0,287 | Negative | Negative | Negative | Negative | Negative | Negative | Negative | Negative | Negative |
| **97** | 0,233 | Negative | Negative | Negative | Negative | Negative | Negative | Negative | Negative | Negative |
| **98** | 0,035 | Negative | Negative | Negative | Negative | Negative | Negative | Negative | Negative | Negative |
| **99** | 0,744 | Negative | Positive | Positive | Positive | Negative | Negative | Negative | Negative | Negative |
| **100** | 0,117 | Negative | Negative | Negative | Negative | Negative | Negative | Negative | Negative | Negative |
| **101** | 0,866 | *Aelurostrongylus abstrusus* | Positive | Positive | Positive | Positive | Negative | Negative | Negative | Negative |
| **102** | 0,204 | *Aelurostrongylus abstrusus* | Negative | Negative | Negative | Negative | Negative | Negative | Negative | Negative |
| **103** | 0,613 | *Aelurostrongylus abstrusus* | Positive | Positive | Negative | Negative | Negative | Negative | Negative | Negative |
| **104** | 0,342 | *Aelurostrongylus abstrusus* | Negative | Negative | Negative | Negative | Negative | Negative | Negative | Negative |
| **105** | 0,118 | *Aelurostrongylus abstrusus* | Negative | Negative | Negative | Negative | Negative | Negative | Negative | Negative |
| **106** | 0,52 | *Aelurostrongylus abstrusus* | Positive | Negative | Negative | Negative | Negative | Negative | Negative | Negative |
| **107** | 0,327 | *Aelurostrongylus abstrusus* | Negative | Negative | Negative | Negative | Negative | Negative | Negative | Negative |
| **108** | 0,043 | *Aelurostrongylus abstrusus* | Negative | Negative | Negative | Negative | Negative | Negative | Negative | Negative |
| **109** | 0,659 | *Aelurostrongylus abstrusus* | Positive | Positive | Negative | Negative | Negative | Negative | Negative | Negative |
| **110** | 0,713 | *Aelurostrongylus abstrusus* | Positive | Positive | Positive | Negative | Negative | Negative | Negative | Negative |
| **111** | 0,169 | *Aelurostrongylus abstrusus* | Negative | Negative | Negative | Negative | Negative | Negative | Negative | Negative |
| **112** | 0,051 | *Aelurostrongylus abstrusus* | Negative | Negative | Negative | Negative | Negative | Negative | Negative | Negative |
| **113** | 0,342 | *Aelurostrongylus abstrusus* | Negative | Negative | Negative | Negative | Negative | Negative | Negative | Negative |
| **114** | 0,242 | *Aelurostrongylus abstrusus* | Negative | Negative | Negative | Negative | Negative | Negative | Negative | Negative |
| **115** | 0,82 | *Aelurostrongylus abstrusus* | Positive | Positive | Positive | Positive | Negative | Negative | Negative | Negative |
| **116** | 0,321 | *Aelurostrongylus abstrusus* | Negative | Negative | Negative | Negative | Negative | Negative | Negative | Negative |
| **117** | 0,697 | *Aelurostrongylus abstrusus* | Positive | Positive | Negative | Negative | Negative | Negative | Negative | Negative |
| **118** | 0,972 | *Aelurostrongylus abstrusus* | Positive | Positive | Positive | Positive | Positive | Negative | Negative | Negative |
| **119** | 0,114 | *Aelurostrongylus abstrusus* | Negative | Negative | Negative | Negative | Negative | Negative | Negative | Negative |
| **120** | 0,52 | *Aelurostrongylus abstrusus* | Positive | Negative | Negative | Negative | Negative | Negative | Negative | Negative |
| **121** | 0,161 | *Taenia* sp. | Negative | Negative | Negative | Negative | Negative | Negative | Negative | Negative |
| **122** | 0,239 | *Toxocara cati* | Negative | Negative | Negative | Negative | Negative | Negative | Negative | Negative |
| **123** | 0,014 | *Toxocara cati* | Negative | Negative | Negative | Negative | Negative | Negative | Negative | Negative |
| **124** | 0,052 | *Toxocara cati* | Negative | Negative | Negative | Negative | Negative | Negative | Negative | Negative |
| **125** | 0,859 | *Toxocara cati* | Positive | Positive | Positive | Positive | Negative | Negative | Negative | Negative |
| **126** | 0,632 | *Taenia* sp. | Positive | Positive | Negative | Negative | Negative | Negative | Negative | Negative |
| **127** | 0,278 | *Taenia* sp. | Negative | Negative | Negative | Negative | Negative | Negative | Negative | Negative |
| **128** | 0,056 | *Taenia* sp. | Negative | Negative | Negative | Negative | Negative | Negative | Negative | Negative |
| **129** | 0,528 | *Taenia* sp. | Positive | Negative | Negative | Negative | Negative | Negative | Negative | Negative |
| **130** | 0,189 | *Toxocara cati* | Negative | Negative | Negative | Negative | Negative | Negative | Negative | Negative |
| **131** | 0,137 | *Toxocara cati* | Negative | Negative | Negative | Negative | Negative | Negative | Negative | Negative |
| **132** | 0,915 | *Taenia* sp. | Positive | Positive | Positive | Positive | Positive | Negative | Negative | Negative |
| **133** | 0,163 | *Mesocestoides lineatus, Taenia spp, Toxocara cati* | Negative | Negative | Negative | Negative | Negative | Negative | Negative | Negative |
| **134** | 0,428 | *Toxocara cati* | Negative | Negative | Negative | Negative | Negative | Negative | Negative | Negative |
| **135** | 0,045 | *Toxocara cati* | Negative | Negative | Negative | Negative | Negative | Negative | Negative | Negative |
| **136** | 0,52 | *Toxocara cati* | Positive | Negative | Negative | Negative | Negative | Negative | Negative | Negative |
| **137** | 0,102 | *Dipylidium caninum* | Negative | Negative | Negative | Negative | Negative | Negative | Negative | Negative |
| **138** | 0,075 | *Dipylidium caninum, Toxocara cati* | Negative | Negative | Negative | Negative | Negative | Negative | Negative | Negative |
| **139** | 0,169 | *Toxocara cati* | Negative | Negative | Negative | Negative | Negative | Negative | Negative | Negative |
| **140** | 0,689 | *Toxocara cati, Taenia* sp. | Positive | Positive | Negative | Negative | Negative | Negative | Negative | Negative |
| **141** | 2 | *Dirofilaria immitis* | Positive | Positive | Positive | Positive | Positive | Positive | Positive | Positive |
| **142** | 1,219 | *Dirofilaria immitis* | Positive | Positive | Positive | Positive | Positive | Positive | Positive | Positive |
| **143** | 1,879 | *Dirofilaria immitis* | Positive | Positive | Positive | Positive | Positive | Positive | Positive | Positive |
| **144** | 1,173 | *Dirofilaria immitis* | Positive | Positive | Positive | Positive | Positive | Positive | Positive | Positive |
| **145** | 1,842 | *Dirofilaria immitis* | Positive | Positive | Positive | Positive | Positive | Positive | Positive | Positive |
| **146** | 1,412 | *Dirofilaria immitis* | Positive | Positive | Positive | Positive | Positive | Positive | Positive | Positive |
| **147** | 1,205 | *Dirofilaria immitis* | Positive | Positive | Positive | Positive | Positive | Positive | Positive | Positive |
| **148** | 1,479 | *Dirofilaria immitis* | Positive | Positive | Positive | Positive | Positive | Positive | Positive | Positive |
| **149** | 1,663 | *Dirofilaria repens* | Positive | Positive | Positive | Positive | Positive | Positive | Positive | Positive |
| **150** | 1,212 | *Dirofilaria repens* | Positive | Positive | Positive | Positive | Positive | Positive | Positive | Positive |
| **151** | 0,982 | *Dirofilaria immitis* | Positive | Positive | Positive | Positive | Positive | Negative | Negative | Negative |

**Table S2.** ELISA positivity according to results retrieved by other *Dirofilaria* spp. tests (i.e., qPCR and SNAP test).

| **Sample ID** | **ELISA** | **SNAP** | **qPCR** |
| --- | --- | --- | --- |
| **141** | Positive | Negative | Positive |
| **142** | Positive | Positive | Negative |
| **143** | Positive | Positive | Negative |
| **144** | Positive | Positive | Negative |
| **145** | Positive | Positive | Negative |
| **146** | Positive | Positive | Positive |
| **147** | Positive | Positive | Negative |
| **148** | Positive | Positive | Positive |
| **149** | Positive | Negative | Positive |
| **150** | Positive | Negative | Positive |
| **151** | Negative | Positive | Negative |
